# Matching the Inhaler to the Patient in COPD

**DOI:** 10.3390/jcm10235683

**Published:** 2021-12-01

**Authors:** Rudi Peché, Darush Attar-Zadeh, Jane Scullion, Janwillem Kocks

**Affiliations:** 1 Department of Pneumology, Centre Hospitalier Universitaire de Charleroi, 6042 Charleroi, Belgium; 2 North Central London Clinical Commissioning Group (CCG), London N11 1GN, UK; darushattar@hotmail.com; 3 Department of Respiratory Medicine, University Hospitals of Leicester NHS Trust, Leicester LE1 5WW, UK; jane.scullion@uhl-tr.nhs.uk; 4 General Practitioners Research Institute, 9713 GH Groningen, The Netherlands; janwillem@gpri.nl; 5 GRIAC Research Institute, University Medical Center Groningen, University of Groningen, 9712 CP Groningen, The Netherlands; 6 Observational and Pragmatic Research Institute, Singapore 409051, Singapore

**Keywords:** primary care, respiratory, inhaler, device, patient

## Abstract

Selecting the most appropriate inhalation device from the wide range available is essential for the successful management of patients with chronic obstructive pulmonary disease. Although choice is good for healthcare professionals, knowing which inhaler to prescribe is a complex consideration. Among the key factors to consider are quality of disease control, inhaler technique, inhaler resistance and inspiratory flow, inhaler design and mechanisms of drug delivery, insurance and reimbursement restrictions, and environmental impact. In this article, we offer a simple, practical tool that brings together all these factors and includes hyperlinks to other published resources from the United Kingdom, Belgium, and The Netherlands.

## 1. Introduction

Chronic obstructive pulmonary disease (COPD) is forecast to become the third-leading cause of death worldwide by 2030. As a preventable and treatable disease, COPD is managed through a combination of pharmacologic therapy, pulmonary rehabilitation, and smoking cessation [1,2]. The Global Initiative for Chronic Obstructive Lung Disease (GOLD) 2021 strategy report recommends initiation of pharmacotherapy based on an individualized assessment of a patient’s exacerbation history, symptom severity, and blood eosinophil count (ABCD classification scheme). Long-acting bronchodilators (long-acting muscarinic antagonists (LAMAs) or long-acting β_2_-agonists (LABAs)), alone or in combination, are the mainstay of COPD treatment, with inhaled corticosteroids (ICS) reserved for patients who are highly symptomatic, with an eosinophil count of ≥300 cells/µL (for patients with one exacerbation in the previous year) or ≥100 cells/µL (for patients with ≥2 moderate exacerbations or ≥1 exacerbation requiring hospitalization in the previous year) [2].

A wide range of inhaler types are available to deliver COPD therapy, each with its own unique features. Although choice is good for the healthcare professional (HCP), knowing which inhaler to prescribe from the many available options is a complex consideration. In this article, we offer a simple, practical tool to help HCPs make their decisions and discuss the different factors to be taken into account. The availability of different inhaler types to use with specific medications is beyond the scope of this review and should be checked locally.

## 2. Overview of Approaches to Match the Inhaler to the Patient

Among the key factors to consider when selecting the most appropriate inhaler for an individual patient are quality of disease control, inhaler technique, inhaler resistance and inspiratory flow, inhaler design and mechanisms of drug delivery, insurance and reimbursement restrictions, and environmental impact—something of growing importance not only to national healthcare systems but also to patients [3,4].

**Figure 1:**
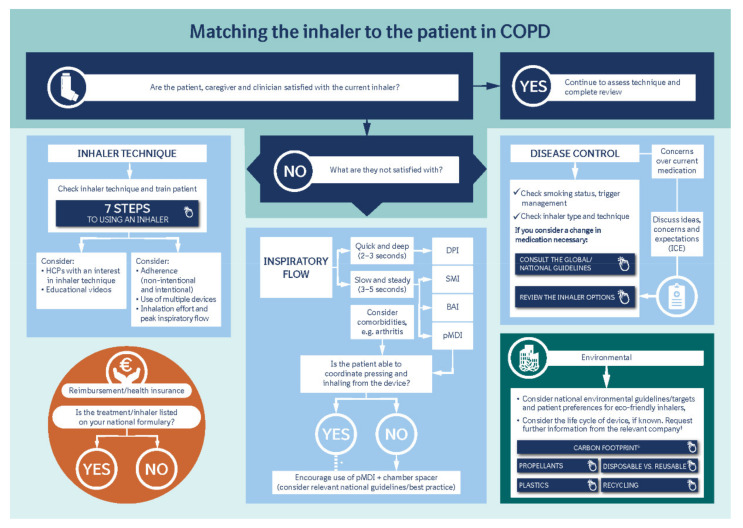
Matching the inhaler to the patient in COPD: ^†^ Take into account the influence of the pharmaceutical industry on sponsored research; ^‡^ Carbon footprint encompasses several elements throughout the product lifecycle, including manufacturing, propellants and plastic use/recycling. BAI, breath-actuated inhaler; DPI, dry powder inhaler; HCP, healthcare provider; ICE, ideas, concerns, and expectations; pMDI: pressurized metered-dose inhaler; SMI, soft mist inhaler. 7 Steps (https://www.respiratoryfutures.org.uk/media/69774/ukig-inhaler-standards-january-2017.pdf; accessed on 25 November 2021); Consult guidelines (https://goldcopd.org/2021-gold-reports/; accessed on 25 November 2021); Review inhaler (https://www.rightbreathe.com/; accessed on 25 November 2021); Carbon footprint (https://bmjopen.bmj.com/content/9/10/e028763; accessed on 25 November 2021); Propellants (https://bmjopenrespres.bmj.com/content/7/1/e000571; accessed on 25 November 2021); Disposable v reusable (https://link.springer.com/article/10.1007/s12325-019-01028-y; accessed on 25 November 2021); Plastics (https://erj.ersjournals.com/content/55/2/2000048; accessed on 25 November 2021); Recycling (https://bjgp.org/content/70/690/30; accessed on 25 November 2021).

To help HCPs with their choice of inhaler types, we have developed a tool that brings together all these factors and which includes hyperlinks to other published resources from the United Kingdom, Belgium, and The Netherlands (Figure 1).

In the following sections, we will describe each of these factors in more detail.

## 3. Disease Control

When a patient presents with poor disease control, inhaler technique is the first thing that should be checked before considering a change of medication (alongside discussion of smoking cessation and other possible disease triggers). If, after checking technique, a change in medication is still considered necessary, HCPs should consult the relevant global/national guidelines [2] and then review the inhaler options available for the patient. Equally, if a patient presents with good disease control, a step-down in therapy can be carefully considered, and selecting a similar inhalation device can help to facilitate a smooth transition. If a patient expresses concerns around their current medication, level of disease control, or technique, it is important for all HCPs to address these in their interactions with patients to improve health-related outcomes. The ‘ideas, concerns, and expectations’ consultation model is a patient-centered approach to prescribing, which the evidence suggests could lead to more positive, conservative decisions in the prescription of medication [5].

## 4. Inhaler Technique

As per the 2021 GOLD strategy report, inhaler technique and adherence should be assessed before concluding that an existing therapy is inadequate [2]. The importance of training in inhaler technique cannot be overstated [2]. Observational studies have clearly demonstrated a link between poor technique and inadequate control of symptoms in patients with COPD [6,7]. On average, over two–thirds of patients make at least one error when using their inhalation device [2], with estimates as high as ~90% in some studies (depending on the inhaler and the type of assessment). A systematic review and meta-analysis of 37 studies identified a range of patient-related and inhaler-related characteristics that are predictive of inhalation technique errors [8]. These included older age, lower educational level, female gender, low socioeconomic status, number of comorbidities and disease severity (patient-related factors), lack of training on device use, a longer overall duration of device use, and the use of multiple inhalers (inhaler-related factors) [8]. Other device errors, such as poor synchronization of pressing and inhalation, and the patient’s ability to inhale for long enough (and hold their breath for long enough), can all impact the deposition of the inhaled drug in the lung, and thus the quality of disease control [9].

Inhaler technique errors may manifest because patients do not feel confident when using their inhaler, due to either never having received instruction or forgetting the instructions they initially received [10,11]. Educating and training the patient regularly can, therefore, improve technique and adherence [12,13,14]. Different stakeholders can be involved in training, such as pulmonologists, general practitioners, respiratory nurses, and pharmacists [14,15,16,17]. In an attempt to bridge the gap between practitioners and patients, the UK Inhaler Group (2016) has published a seven-step guide to using an inhaler device correctly, which is relevant for all inhalers [18]. It is worth noting that even if the actuation of an inhaler contains an optimal dose of effective medication, a patient may still have poor clinical outcomes if their inhalation technique is not correct.

It is also important to differentiate between intentional and non-intentional non-adherence [12]. Some patients choose not to use their inhaler—due to fear of side effects, for example—which negatively impacts their disease control. Other patients actively engage with disease management but may not receive the full benefits of their medication due to incorrect inhalation technique, or other reasons beyond their control. For example, comorbidities such as arthritis can impact the handling of an inhaler. Ciciliani et al. assessed finger strength across different inhaler types, demonstrating that different finger strengths are required for each device, and therefore concluding that a patient’s manual dexterity and strength need to be taken into consideration when prescribing a device [19].

Another important aspect to consider is device familiarity, as previous studies have demonstrated that patients with COPD who were prescribed inhalation devices requiring a similar technique to their previous devices had better outcomes (such as a lower rate of exacerbations) than patients who were prescribed devices that required a different inhalation technique [20]. In reality, however, some patients may have to overcome the potential confusion associated with learning more than one inhaler technique. Short-acting reliever inhalers, for example, which are typically pressurized metered-dose inhalers (pMDIs), often utilized due to cost or the potential for more effective delivery of bronchodilation in an emergency, may require a different inhalation technique from the inhaler or inhalers used to deliver a patient’s long-acting maintenance therapy.

## 5. Inspiratory Flow and Aerosol Deposition

The velocity of the particles from the inhalation device, and the impact they have on the oropharynx and larynx, are determined to some extent by the patient’s inspiratory flow, which can be measured in the clinic [21]. To optimize delivery of the drug to the lungs, patients must be able to generate inspiratory flow at an appropriate speed and duration for each inhaler type. Some devices require a quick and deep inhalation, whereas others require a slow and steady inhalation. When using a pMDI, for example, fast inspiration is not recommended due to the turbulent airflow and fast particle velocity that this causes, which can increase drug deposition in the oropharynx and upper airways [22]. Most dry powder inhalers (DPIs), by contrast, require a quick and deep inspiratory flow to separate the drug particles from their carrier molecules [22]. Soft mist inhalers (SMIs) generate a slow-moving mist of drug [23], and slow and steady inhalation is therefore required for optimal delivery to the lungs.

**Figure 2:**
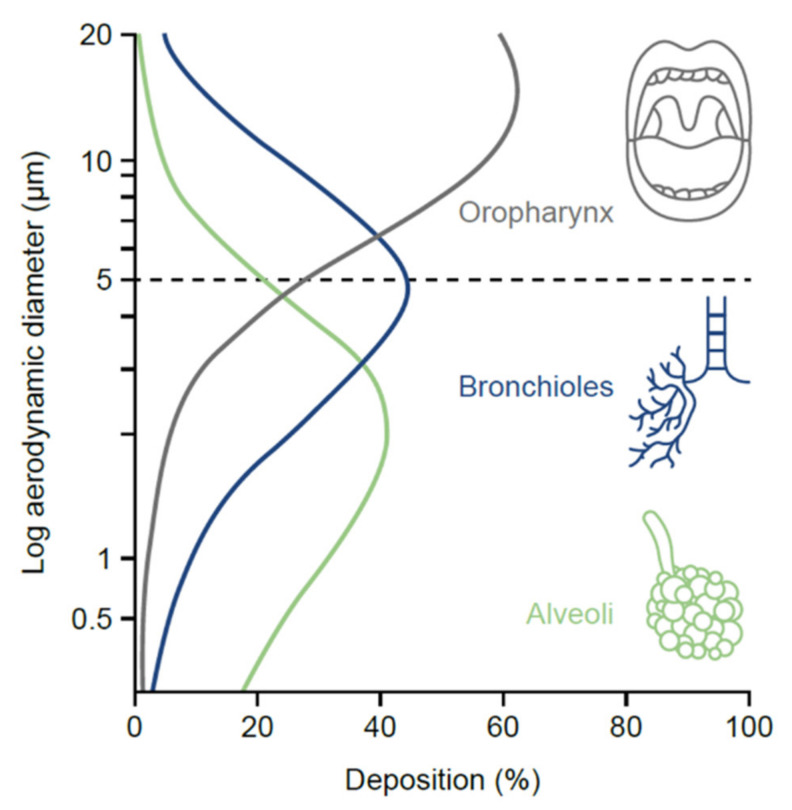
Relationship between aerodynamic particle diameter and drug deposition in the upper and lower airways.

The lungs provide a vast surface area and low enzymatic activity for more targeted drug delivery and systemic absorption [24]. Unlike oral or intravenous therapy, aerosolized therapy distributes the drug directly to the internal lumen of the airways, resulting in a lower systemic concentration of therapy compared with parenteral treatment, and quicker bronchodilation [17]. For an inhalation device to successfully deliver a drug to the lungs, it needs to contain particles of an appropriate size that can penetrate the upper and lower airways (Figure 2) [17].

One of the most important particle-related factors that affects aerosol deposition is aerodynamic diameter. This is often referred to in terms of mass median aerodynamic diameter, i.e., the diameter at which 50% of the particles of an aerosol are larger by mass and 50% are smaller [17]. Particles <5 µm in diameter (fine-particle fraction) have the greatest potential to deposit in the lungs, penetrating beyond the upper airways and traveling to the peripheral airways and alveoli (the majority of devices now generate a significant proportion of particles ≤5 µm to ensure optimal drug delivery to the lungs) [17]. As the particles reach the lung periphery, the rate of airflow decreases, increasing drug deposition. If a patient is able to hold their breath for longer than 5 s after inhalation, the drug particles will spend a longer time in the peripheral airways undisturbed by inspiratory or expiratory flow, resulting in higher deposition [25].

## 6. Inhaler Design and Mechanisms of Drug Delivery

**Figure 3:**
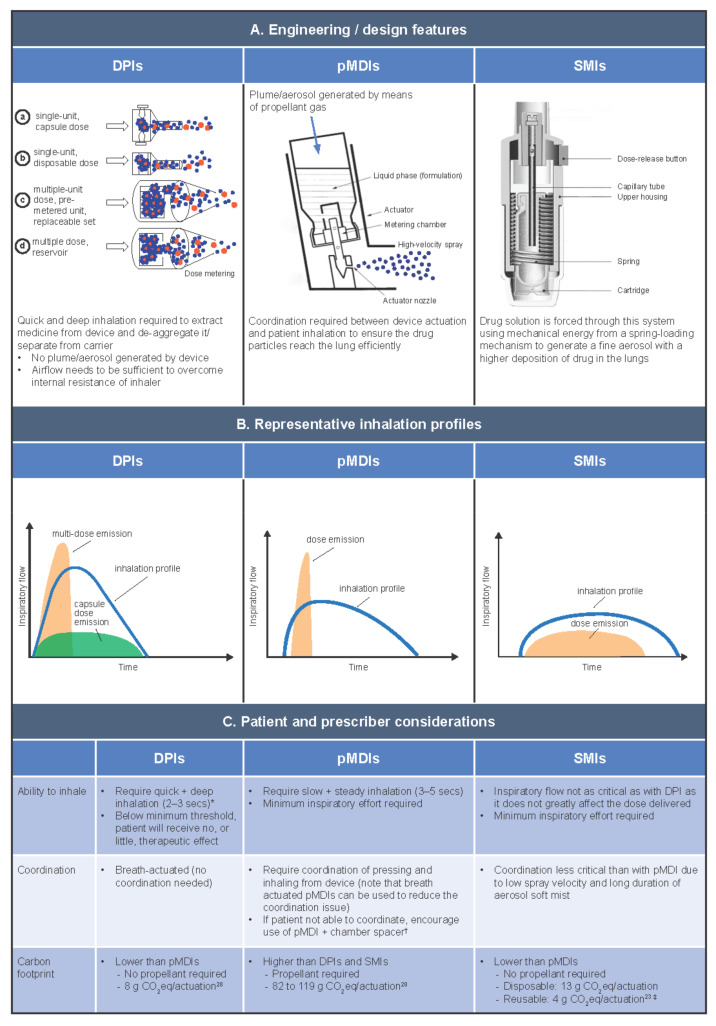
Design features, inhalation profiles, and patient considerations for the three main classes of inhalation device. * For low resistance DPIs, slow and deep inhalation is required; ^†^ Consider relevant national guidelines/best practice; ^‡^ The disposable device provides one month’s usage (60 actuations); the reusable device, which has replaceable cartridges, provides six months’ usage (360 actuations). DPI, dry powder inhaler; pMDI, pressurized metered-dose inhaler; SMI, soft mist inhaler. [28].

Understanding the different mechanisms of drug delivery, and how these are affected by patient airflow profiles, can help HCPs select the right device for their patient from the three main classes of inhalation device—pMDIs, DPIs, and SMIs (Figure 3) [26]. A nebulizer is a device that is also used in some countries for inhaled drug delivery. However, they have many disadvantages, such as restricted portability, lack of chemical stability, and longer treatment times, and they can result in a significant waste of medication [27]. They are not recommended for the management of patients with COPD [2]; therefore, they will not be discussed further within this review.

### 6.1. DPIs

DPIs are portable and compact, and unlike pMDIs, they do not contain propellant gas. There are two basic types of DPI: multidose DPIs and single-dose capsule DPIs. There are also two variations of the multi-dose DPI: those containing a bulk formulation in a reservoir, where the meter is patient controlled; and those containing pre-metered, factory-dispensed doses packaged inside blisters within the device [17], where the meter is not patient controlled. The inspiratory force and airflow generated by the patient when they inhale from a DPI de-aggregates the drug formulation from its carrier molecules, creating the energy to extract the drug from the device and deliver it into the patient. DPIs, therefore, have a minimum threshold of optimum flow, and if the inspiratory flow is too low, a reduced dose is delivered, and the patient may receive sub-optimal clinical benefit [29]. Unlike pMDIs, a patient’s inhalation flow profile when using a DPI is typically quick (>60 L/min) or, depending on the resistance of the device, slightly slower (e.g., >30 L/min) [30]. Quicker inhalation is particularly critical for reservoir/blister-type DPIs, which emit the dose earlier compared with capsule DPIs [17]. Regardless of speed, the inhalation from a DPI must be deep to achieve optimal drug delivery [17]. New smaller-particle formulations have been developed for use with DPIs, which may help to optimize drug delivery [31].

As DPIs require a minimum level of inspiratory flow to separate the medication from its carrier molecule, they are less appropriate for use during an exacerbation [26]. Studies suggest that failure to achieve forceful inspiratory flow through the device is a common mishandling error with DPIs, occurring in an estimated 26–38% of cases [7,31,32] and impacting clinical outcomes [7]. In a systematic review of inhaler use errors by Sanchis et al., other common errors associated with DPI use were found to be lack of full expiration before inhalation (42–50%), lack of post-inhalation breath-hold (33–40%), and incorrect device preparation (26–33%) [32].

### 6.2. pMDIs

pMDIs are compact, contain a metered dose, and can be used even in very breathless patients (for example, during exacerbations); however, they require good hand–lung coordination, which some patients can find challenging. pMDIs have a higher carbon footprint compared with other devices [23,28], due to the global warming potential of the propellant gas they contain (originally chlorofluorocarbon (CFC), later replaced with hydrofluorocarbon), which aerosolizes the drug formulation to produce a high-velocity spray. The use of propellants and the production of the high-velocity spray mean that inhalation from pMDIs must be well timed and well controlled (slow and steady), otherwise the spray may be deposited largely in the mouth or throat. Breath-actuated pMDIs have been developed as an alternative to traditional pMDIs to avoid the problem of poor actuation–inhalation coordination by automatically actuating at low inspiratory flow rates [17]. Training devices that do not contain any medication are also available to improve patients’ pMDI technique alongside verbal inhaler training by HCPs [33]. CFC-containing pMDIs previously caused the freon effect (chilling sensation at the back of the throat after inhalation), which negatively impacted patient inhalation. A small amount of ethanol has since been added to pMDI formulations to alter the taste, increase the temperature, and decrease the velocity at which the medication is released from the device [17].

Even with the right inhalation technique, pMDIs can leave a high deposition of the drug in the patient’s mouth and oropharynx. Spacers can help to alleviate the difficulty of inhaling while pressing for users of pMDIs. As many patients struggle with this technique, HCPs should actively encourage the use of a spacer (in The Netherlands, for example, it is routine practice for all pMDIs to be prescribed with a spacer). The advantages of using a spacer are that the inspiratory effort of the patient is not as critical, and the patient is likely to receive a higher lung deposition than when using a pMDI alone (i.e., less deposition in the mouth and oropharynx). However, spacers are less portable, bring additional cost, and require the correct maintenance [26]. Note that pMDIs that emit smaller particles are associated with improved lung deposition and lower oropharyngeal deposition without the need for a spacer and provide an alternative option compared with traditional pMDIs [34].

### 6.3. SMIs

SMIs are also portable and compact. In addition, they have a lower dependency on the inspiratory effort of the patient to deliver a high fine-particle fraction and high lung deposition, and do not contain propellants [26]. SMIs atomize the drug solution using mechanical energy imparted by pressure from a spring. When the pressure is released, the solution is forced through a fine nozzle, producing a slow-moving mist of the drug formulation. SMIs deliver the high fine-particle fraction at slow velocity, resulting in good lung deposition even when a patient has a low inhalation flow [35]. As such, it is not necessary for physicians to consider peak inspiratory flow when prescribing an SMI, as the patient’s inspiratory capacity does not affect the dose delivered. There is only one SMI device currently available, which is compatible with a limited range of medications relative to other inhaler types [2,26]. Thus, it is important to consider the medication options and their compatibility with available devices alongside other factors (such as patient skills and cost) when choosing an inhaler [26].

## 7. Reimbursement

Once the most appropriate device has been selected, it is important to assess if there are any restrictions associated with insurance, commissioning, or reimbursement. HCPs may not always have full autonomy in selecting a device across Europe, and these influences are country specific. The HCP will need to check if, after a full assessment of the patient’s needs, the treatment they are wishing to prescribe is listed on their local or national formulary; if it is not, they may need to adapt their selection. Due to an increasing number of devices available to HCPs, the use of local formularies (preferred prescribing lists) would be beneficial, as long as appropriate inhalation devices are available. This will help ensure that prescribing is appropriate within licensed or local recommendations.

## 8. Environmental Impact

The production of pharmaceutical products is a process that uses a vast amount of resources, which inevitably has a significant impact on the environment. All inhalation devices have a life cycle, and some pharmaceutical companies are assessing the life cycles of their products in an attempt to minimize their environmental impact. Measures such as reduction of hazardous substances, introduction of energy-efficient measures within the manufacturing process, and implementation of eco-designs in product development aim to lessen the environmental effects of their products [36].

Environmental considerations are complex, and it is difficult to account for all aspects in this article. However, national environmental guidelines and targets, and patient preference for eco-friendly inhalers, as well as the carbon footprint of DPIs, pMDIs, and SMIs, should be considered within the device selection process. In the United Kingdom, for example, the National Health Service aims to change to low-carbon inhalers over the long term to enable a 4% reduction in their carbon footprint. Due to the propellant they contain, pMDIs have a higher carbon footprint than DPIs and SMIs, thus contributing more to global warming [23,28]. For this reason, the British Thoracic Society recommends changing from pMDIs to DPIs or SMIs, as low-carbon, propellant-free alternatives, whenever they are likely to be equally effective and patients are able to use them safely. It is also important that we optimize the recycling of plastic from inhalers (through pharmacy-led inhaler recycling schemes, for example) both to address the environmental problem of plastic waste and to reduce the carbon footprint associated with excess plastic manufacture and disposal [37]. A further reduction in carbon footprint is associated with re-usable versus single-use devices. For example, a re-usable device can reduce the carbon footprint of SMIs by 71% over 6 months [23]. However, no changes in device should be made unless both the patient and clinician are happy. Shared decision-making is a key part of greener healthcare, and ultimately, the most appropriate inhaler is the device that the patient can and will use.

## 9. Conclusions

Although the multitude of inhalation devices allows for a wide range of choice, there are many factors to take into consideration when matching patients to the most suitable device. Factors such as quality of disease control, inhaler technique, inhaler resistance and inspiratory flow, insurance and reimbursement restrictions, and environmental impact all need to be considered. In addition, a greater understanding of how each inhalation device works and, therefore, how drug formulations are delivered to the patient, will help HCPs match their patients to the right inhaler.
